# Relevance of SIRT1-NF-κB Axis as Therapeutic Target to Ameliorate Inflammation in Liver Disease

**DOI:** 10.3390/ijms21113858

**Published:** 2020-05-29

**Authors:** Estefanía de Gregorio, Anna Colell, Albert Morales, Montserrat Marí

**Affiliations:** 1 Department of Cell Death and Proliferation, IIBB-CSIC, IDIBAPS, 08036 Barcelona, Spain; estefania.degregorio@iibb.csic.es; 2 Department of Cell Death and Proliferation, IIBB-CSIC, IDIBAPS, Centro de Investigación Biomédica en Red sobre Enfermedades Neurodegenerativas (CIBERNED), 08036 Barcelona, Spain; anna.colell@iibb.csic.es; 3 Department of Cell Death and Proliferation, IIBB-CSIC, Barcelona Clinic Liver Cancer Group, Liver Unit, Hospital Clínic of Barcelona, University of Barcelona, CIBEREHD, 08036 Barcelona, Spain; amorales@clinic.cat

**Keywords:** SIRT1, NF-κB, inflammation, liver, NAFLD, cathepsins, AMPK, PPAR, NAD^+^, STACs

## Abstract

Inflammation is an adaptive response in pursuit of homeostasis reestablishment triggered by harmful conditions or stimuli, such as an infection or tissue damage. Liver diseases cause approximately 2 million deaths per year worldwide and hepatic inflammation is a common factor to all of them, being the main driver of hepatic tissue damage and causing progression from non-alcoholic fatty liver disease (NAFLD) to non-alcoholic steatohepatitis (NASH), cirrhosis and, ultimately, hepatocellular carcinoma (HCC). The metabolic sensor SIRT1, a class III histone deacetylase with strong expression in metabolic tissues such as the liver, and transcription factor NF-κB, a master regulator of inflammatory response, show an antagonistic relationship in controlling inflammation. For this reason, SIRT1 targeting is emerging as a potential strategy to improve different metabolic and/or inflammatory pathologies. In this review, we explore diverse upstream regulators and some natural/synthetic activators of SIRT1 as possible therapeutic treatment for liver diseases.

## 1. Introduction

Sirtuins are a family of class III histone deacetylases (HDAC) distinguished by possessing a catalytic activity dependent on nicotinamide adenine dinucleotide (NAD^+^) cellular availability and, therefore, being regulated by NAD^+^/NADH cellular ratio [1]. In mammals, this family includes seven members (SIRT1-SIRT7) [2] and among them, Sirtuin 1 (SIRT1) has been the most studied.

SIRT1 has been considered as a cellular metabolic sensor due to its ability to couple the metabolic status of the cell (NAD^+^ availability) to chromatin structure [3] and, hence, to gene transcription, through modification of histones and non-histone proteins [4]. The non-histone protein targets of SIRT1 are diverse (eg. p53, FOXO, PGC1-α, NF-κB, PARP1…) [5,6,7,8,9], and their modifications result in different outcomes in the cell, such as apoptosis, stress oxidative response, mitochondrial biogenesis and inflammatory response, among others [10]. Regarding its location, SIRT1 resides mostly in the nucleus, but can shuttle from this organelle to the cytosol through its two nuclear localization signals (NLS) and its two nuclear exportation signals (NES) [11]. Moreover, subcellular localization of SIRT1 may change depending on cell type, tissue and in response to physiological and pathological stimuli [11].

SIRT1 has been involved in numerous metabolic pathways, such as gluconeogenesis, glycolysis, fatty acid oxidation and synthesis, oxidative phosphorylation or urea cycle [12,13], and in several fundamental and homeostatic processes like mitochondrial biogenesis, inflammation, apoptosis [14] or tumorigenesis [5]. The role of SIRT1 in cancer comes from its association with p53, its first known non-histone substrate. However, recent studies suggest that SIRT1 may act as a tumor suppressor or tumor promoter, depending on SIRT1 localization and cell type [5]. Consequently, SIRT1 expression and regulation has been described in different organs including adipose tissue, pancreas, brain, muscle or liver [14]. 

## 2. SIRT1 in Liver Metabolism

In the liver, SIRT1 partially regulates glucose, lipids and cholesterol metabolism. Of note, changes in the concentration of nutrients and hormones during fasting/intake periods control the expression of SIRT1 [15].

During fasting, there is an initial increase in glucagon levels, produced by pancreatic alpha cells, which leads to a rise in gene transcription of both SIRT1 and gluconeogenesis genes in the liver, through the cyclic AMP response element-binding protein (CREB) and its co-activator protein, CREB-regulated transcription coactivator 2 (CRTC2) [12,15]. Gluconeogenesis is an anabolic pathway of metabolism that allows glucose biosynthesis from different sources: glucogenic amino acids, lactate, glycerol or tricarboxylic acid (TCA) cycle intermediates [16]. If fasting is prolonged, SIRT1 first deacetylates CRTC2 protein, which results in its targeting for ubiquitinization and degradation by the proteasome. Secondly, SIRT1 deacetylates peroxisome proliferator-activated receptor γ coactivator 1-α (PGC1-α) and forkhead box O1 (FOXO1), key participants in β-oxidation and gluconeogenesis regulation, increasing their transcriptional activity [17]. On the one hand, deacetylation and activation of PGC1-α by SIRT1 results in increased fatty acid oxidation and improved glucose homeostasis [18]. On the other hand, the activation of FOXO1, by its deacetylation by SIRT1, increases gluconeogenesis [19]. In this way, the maintenance of both metabolic processes can supply the body’s energy needs during prolonged fasting. In contrast, under nutrient intake conditions, carbohydrate-responsive element-binding protein (ChREBP) transcription factor, induced by circulating high glucose and fatty acids levels, represses the expression of SIRT1 [20].

SIRT1 not only regulates glucose metabolism in the liver, but also lipids and cholesterol homeostasis. During fasting, free fatty acids are released from adipose tissue and subjected to β-oxidation in the liver to provide energy [16]. By contrast, under fed conditions, liver synthesizes fatty acids (lipogenesis), which are then stored in adipose tissue [16]. In a starving state, SIRT1 promotes fatty acid oxidation by activating peroxisome proliferator-activated receptor α (PPAR-α) [12]. PPAR-α is a transcription factor able to bind fatty acids, and whose union unleashes an increase in expression of genes related to fatty acid catabolism in the mitochondrial matrix [12]. SIRT1 enhances PPAR-α activation by deacetylating the co-activator of PPAR-α: PGC1-α [12]. Additionally, SIRT1 deacetylates sterol regulatory element-binding protein 1 (SREBP1) transcription factor, targeting it for degradation via ubiquitin/proteasome system, which results in hepatic repression of lipids and cholesterol synthesis [21]. SIRT1 also facilitates the action of oxysterols liver X receptor α (LXR-α), whose target gene, ATP-binding cassette transporter A1 (ABCA1), is responsible for high-density lipoprotein (HDL) particle synthesis and reverse cholesterol transport, from peripheral tissues to liver, where it can be secreted into bile [22]. Finally, SIRT1 also regulates cholesterol homeostasis via farnesoid X receptor (FXR), important for bile acids biosynthesis and cholesterol catabolic pathways. Deacetylation of FXR by SIRT1 produces, on the one hand, receptor activation, increasing bile acid synthesis and, on the other hand, it has a positive feedback effect over SIRT1 transcription [23]. 

## 3. SIRT1 in a Liver Metabolic Disorder: NAFLD (Non-Alcoholic Fatty Liver Disease)

Non-alcoholic fatty liver disease (NAFLD) is considered as the hepatic evidence of the metabolic syndrome [24], being the most common liver disease in Western countries. NAFLD affects 17–46% of population, depending on diagnostic method, age, sex and ethnicity [25]. It is defined as the presence of at least 5% of hepatic steatosis, in the absence of secondary causes that lead to an accumulation of intrahepatic lipids, such as an excessive alcohol consumption, congenital liver disorders or long-term treatment with medication that induce steatosis as side effect. In addition, it is frequently associated with metabolic disorders, such as obesity, diabetes mellitus or dyslipidemia [26]. If unresolved, NAFLD may evolve into non-alcoholic steatohepatitis (NASH), cirrhosis and ultimately, hepatocellular carcinoma (HCC) [24]. Currently, there is no approved drug for the treatment of NAFLD, although randomized control trials are being conducted with some drugs that have shown improvement in the regression of hepatic necro-inflammation and/or fibrosis [25,27].

The pathology of the disease courses with excessive initial deposition of triglyceride in the form of lipid droplets within the hepatocytes. This deposition is caused by an imbalance in the control of liver lipids which, in turn, is caused by an increase of fatty acids/triglycerides uptake and/or enhanced de novo lipogenesis, together with an impaired fatty acids β-oxidation, and/or decreased export through very low-density lipoprotein (VLDL) particles [28]. 

Steatosis, entails the activation of the transcription factor nuclear factor kappa B (NF-κB), with the consequent production of pro-inflammatory cytokines such as tumor necrosis factor α (TNF-α), IL6 or IL1β. The production of these cytokines triggers the recruitment and activation of immune system cells that influence inflammation and also promotes the creation of hepatic insulin resistance. Moreover, excess intrahepatic lipids induce lipotoxicity and lead to mitochondrial dysfunction and endoplasmic reticulum stress. In turn, mitochondrial dysfunction favors the production of reactive oxygen species (ROS) causing oxidative stress that eventually damages hepatocytes [29].

The role of sirtuins in NAFLD and other metabolic diseases has been described in both, animals and patient studies. Some in vivo studies with animals with various genetic modifications on hepatic *Sirt1* gene have highlighted the importance of this protein at a metabolic level. Among them, Rodgers et al. [30] reported that a liver-specific SIRT1 knockout (KO) mice fed with a standard-diet presented increased systemic glucose levels together with insulin sensitivity, decreased glucose production and increased hepatic free fatty acid and cholesterol content, among other effects. Meanwhile, Wang et al. [31] described a very similar result with a liver-specific SIRT1 KO (exons 5 and 6 deleted) that developed fatty liver under a normal feeding condition starting at two months of age. Moreover, Purushotam et al. [32] observed that mice with a specific deletion of *Sirt1* gene in hepatocytes resulted in animals presenting a damaged β-oxidation due to an impaired PPAR-α/PGC1-α pathway, developing liver steatosis, inflammation and endoplasmic reticulum stress when fed with a high fat diet.

Likewise, a study with patients with NAFLD have revealed a lower expression of several sirtuins, including SIRT1, and an increase of expression of lipogenic proteins, such as SREBP1, acetyl-CoA carboxylase (ACC) and fatty acid synthase (FAS) in comparison with the control group [33]. Furthermore, another study with patients performed by Mariani et al. [34] evaluating the relationship between serum SIRT1 levels and the degree of liver steatosis in obese patients, showed that SIRT1 levels were quite low in patients with severe hepatic steatosis when compared to obese patients with mild hepatic steatosis. Similarly, serum levels of SIRT1 were inferior in the group of obese patients as compared to those in lean patients. 

## 4. SIRT1 in NF-κB Mediated Inflammation

Inflammation is an adaptive response aimed at restoring homeostasis altered by harmful stimuli, such as infection or tissue damage [35]. During the inflammatory response, several phases develop, starting with an initial pro-inflammatory phase, passing through the adaptive phase and ending with the reinstatement of homeostasis [35]. The switch between the pro-inflammatory and adaptive phase requires a metabolic change from an anabolic state to a catabolic state that depends on the sensing of adenosine monophosphate (AMP) and NAD^+^ levels by AMP-activated protein kinase (AMPK) and sirtuins, respectively. In this way, AMPK and sirtuins are able to couple inflammation and metabolism with chromatin state and gene transcription [36]. 

The nuclear factor kappa B (NF-κB) is a family of inducible transcription factors present in numerous cell types and integrated by seven different members, which form homo and heterodimers: NF-κB1 (p105 and p50), NF-κB2 (p100 and p52), RelA (p65), RelB and c-Rel [37]. NF-κB is considered as a major regulator of the inflammatory response due to its ability to regulate the transcription of genes involved in the establishment of immune and inflammatory response [37,38]. Its regulation occurs at several levels and, to date, three ways have been identified for NF-κB activation: (1) the canonical one, triggered mainly by cytokines such as TNF-α or IL1, and by toll-like receptor (TLR) agonists; (2) the non-canonical one, with an important function in B lymphocytes and (3) the activation induced by DNA damage [39,40]. A second level of regulation is post-translational modifications of NF-κB subunits, carried out by various proteins, including the IκB kinase (IKK) complex. Some of these modifications include processes of phosphorylation, acetylation, ubiquitination and prolyl isomerization, which regulates NF-κB activity by modulating its nuclear translocation, DNA binding, transactivation and interaction with CBP/p300-interacting transactivator 1 [41].

In quiescent cells, NF-κB is located in the cytoplasm, associated with inhibitory proteins (IκB-α, IκB-β, IκB-γ, IκBNS, Bcl-3) and some precursor proteins such as p100 and p105 (which, once cleaved, give rise to p52 and p50 subunits, respectively) [40]. In the canonical activation pathway, upon arrival of a stimulus to the cell, a phosphorylation occurs, followed by ubiquitination and degradation of its inhibitory proteins, in a proteasome dependent-manner. This releases NF-κB, which is then translocated to the nucleus, where it functions by activating gene transcription [42]. 

Both, NF-κB and SIRT1 signaling pathways are evolutionarily conserved mechanisms for the maintenance of homeostasis and whose interaction allows energy balance to be coupled with the immune/inflammatory response [43]. However, the nature of this relationship is antagonistic, so that SIRT1 is capable of inhibiting NF-κB signaling, and vice versa. This antagonism is explained based on two reasons. On the one hand, the body needs to adapt the metabolism to a rapid energy generation system that allows it to respond quickly to a harmful stimulus (such as an infection or tissue damage). On the other hand, it is necessary to re-establish homeostasis conditions once the harmful stimulus has disappeared [43]. Failure to resolve the inflammation would lead to a chronic inflammatory condition, typical of chronic liver diseases [44]. 

A direct association between SIRT1 and RelA/p65 subunit of NF-κB has been described: SIRT1 is able to deacetylate lysine 310 of RelA/p65 subunit, affecting its transcriptional activity and decreasing expression of its anti-apoptotic and pro-inflammatory target genes [45]. Additionally, deacetylation of RelA/p65 at lysine 310 facilitates methylation at lysines 314 and 315, which is important for the ubiquitination and degradation of RelA/p65 [46,47]. The different acetylations/ deacetylations of RelA/p65 can have various effects on NF-κB regulation but, particularly, deacetylation of RelA/p65 by SIRT1 favors the association of p65/p50 complex (the most abundant heterodimer of NF-κB [39,46,47]) with IκB-α (an inhibitor of NF-κB). This association triggers the transport of the NF-κB complex from the nucleus back to the cytoplasm and, therefore, inactivates the activity of NF-κB [48]. Furthermore, several authors have observed the possibility of forming complexes between PGC1-α/PPARs and NF-κB, enhanced by SIRT1, triggering repressive effects on the development of the inflammatory response (reviewed by Kauppinen et al. [43]).

Interestingly, a possible regulatory action of NF-κB on SIRT1 has also been suggested, since regions flanking the SIRT1 gene, both in mice and humans, contain numerous NF-κB binding elements [49,50]. In fact, some authors have already described this possible interaction. For example, Yamakuchi et al. [51] showed that the microRNA 34a (miR-34a) inhibits the expression of SIRT1 by binding to its 3′ UTR region; and Li et al. [52] described the mechanism by which NF-κB, through binding to the promoter region of miR-34a, is able to increase its level of expression. It should be noted that another miR-34a-controlled gene is AXL, a tyrosine kinase receptor that our group has implicated in the development of liver fibrosis [53], particularly in experimental NASH models and patients [54]. A link between AXL expression and SIRT1 has recently been reported in tissue macrophages [55] and may provide new targets for clinical treatment. Whether SIRT1/AXL can act in a coordinated manner and play a role in the progression of chronic liver disease is an aspect that deserves further studies.

Moreover, some factors, as oxidative stress or interferon γ (IFN-γ), can also suppress SIRT1 transcription or activity [52,56,57]. At the same time, NF-κB could induce oxidative stress through the enhancement of expression of ROS generating enzymes, such as NADPH oxidase (NOX) [58,59]. Additionally, it seems that NF-κB could interact with IFN-γ promoter [60]. Similarly, another study demonstrated that another microRNA, miR-378, is a key player in modulating NASH via TNF-α signaling. In particular, miR-378 acts as an important component of the molecular circuit composed by miR-378, AMPK, SIRT1, NF-κB and TNF-α to induce spontaneous activation of inflammatory genes with potential implications in NASH pathogenesis [61]. 

## 5. Hepatic Inflammation

Liver diseases cause approximately 2 million deaths per year worldwide, of which 50% are due to complications of cirrhosis and the other 50% are due to viral hepatitis and HCC, with some differences in burden according to the geographic region, race, gender, ethnicity and socioeconomic strata [62]. One of the most important common triggers of liver diseases is hepatic inflammation.

Hepatic inflammation is an essential part of the wound-healing response of this organ to certain noxious stimulus such as an excessive alcohol consumption, viral hepatitis, excessive fat intake or cholestasis presence, among others [41]. In fact, liver inflammation has the ultimate goal of protecting the hepatocyte from a harmful stimulus, repairing liver tissue and promoting the restoration of homeostasis [63].

For a limited period of time, inflammation results beneficial in helping to combat a possible threat or repair the damage caused by it. However, when inflammation gets chronic it can lead to an irreversible damage to liver parenchyma as a consequence of an excessive secretion of pro-inflammatory cytokines by non-parenchymal liver cells such as Kupffer cells (KCs), liver resident macrophages, or another type of infiltrated immune cells that can induce apoptosis or necrosis in hepatocytes [41].

In turn, both the presence of apoptotic bodies coming from damaged hepatocytes and the presence of certain molecules and inflammatory mediators produced by immune system cells participate in inducing an inflammatory state. In this fashion, ROS, acetaldehyde, compounds resulting from lipid peroxidation or cytokines such as TNF-α, IL1β or transforming growth factor β (TGF-β) may induce activation and proliferation of hepatic stellate cells (HSCs), the main cells responsible for extracellular matrix generation and, therefore, for give rise to liver fibrosis [64]. Moreover, hepatocytes are characterized by possessing an elevated capacity of replication, which allows them to repair and replace damaged tissue, leading chronic inflammation to increase the risk of suffering hepatic carcinogenesis [65].

### 5.1. NF-κB in Hepatocytes: Survival and Proliferation

As previously mentioned, NF-κB has been related to a large number of processes at cellular (adhesion, apoptosis, etc.) and physiological (angiogenesis, inflammation, control of the innate immune response, etc.) level [66]. In the liver, NF-κB relevance in the proliferation/death of hepatocytes by stimulation with TNF-α has been described [66,67,68]. Bacterial derivatives such as LPS (lipopolysaccharide) or cytokines generated in response to these bacterial derivatives, such as TNF-α, are toxic to the hepatocyte [41]. However, in the liver, TNF-α does not usually induce cell death probably due to NF-κB anti-apoptotic response, ensuring hepatocyte viability while the appropriate inflammatory and immune response are initiated [41], and promoting regeneration of hepatocyte mass by stimulating its proliferation facing a possible damage [69]. Proof of the relevance of NF-κB to hepatocyte survival was already provided many years ago by Beg et al. [70], when they showed, in an in vivo KO mouse, that the absence of the p65 subunit of NF-κB caused embryonic death by massive apoptosis of hepatocytes. A few years later, it was demonstrated that this massive loss of hepatocytes was due to the presence of TNF-α, since double TNF/p65 KO mice turned out to be viable and presented a normal liver [71].

The anti-apoptotic actions of NF-κB in hepatocytes are mainly caused by increased transcription of anti-apoptotic genes, including cellular inhibitors of apoptosis (cIAPs), x-linked inhibitor of apoptosis protein (XIAP), B-cell lymphoma (Bcl-2 family member A1), B-cell lymphoma-extra-large (Bcl-XL), cellular FLICE-like inhibitory protein (cFLIP), TNF associated receptor associated factor 1 (TRAF1), TNF receptor associated factor 2 (TRAF2) and growth arrest and DNA damage inducible 45β (GADD45β). Moreover, some stress-related pathways, such as c-Jun-(N)-terminal kinase (JNK) and p38 mitogen-activated protein kinase (MAPK) signaling cascades, were also shown to participate in anti-apoptotic NF-κB-mediated effects [72]. 

Interestingly, and contrary to most publications defending the relevance of NF-κB in hepatocyte survival, some authors have also reported a pro-apoptotic role of NF-κB in the hepatocyte. Luedde et al. [73] showed in a murine model of partial ischemia/reperfusion injury (I/R), that conditional deletion of the subunit IKK2 of IKK complex in hepatocytes entailed a deficient activation of NF-κB and this, in turn, attenuated liver necrosis and inflammation in comparison to wild-type mouse. In the same study, the use of an IKK2 inhibitor was able to protect the mouse from damage caused by I/R injury without sensitizing them to TNF-induced apoptosis. 

Nevertheless, the beneficial function of NF-κB in protecting hepatocytes against TNF-induced apoptosis could also promote the survival of transformed hepatocytes, in this way, supporting malignancy and cancer progression [74]. It is estimated that approximately 80% of HCCs develop in fibrotic or cirrhotic livers, characterized by the existence of chronic damage and inflammation [37]. Activation of NF-κB is known to occur frequently and early in human liver cancers, both of viral and non-viral etiology, and that such activation is associated with the acquisition of a transformed phenotype during hepatocarcinogenesis [41]. 

A chronic infection with the hepatitis B/C virus results in the death of hepatocytes and this, consequently, generates infiltration of immune cells and inflammation. In addition to the death of hepatocytes by the virus infection itself, there is the death of the infected hepatocytes by the action of immune system cells. This triggers compensatory mechanisms to repair liver mass loss through regeneration and repair processes that could eventually lead to the generation of fibrosis and cirrhosis, which in turn can generate HCC [37]. Although the mutations and intracellular signaling pathways involved in the development of HCC from livers infected with the hepatitis B/C virus are still being deciphered, it is known that the activation of NF-κB has a relevant role in this development. An example of this is reported by Kim et al. [75] in which they described the participation of the hepatitis B viral X (HBx) protein, capable of activating NF-κB by upregulating of TANK-binding kinase 1 (TBK1) protein. Regarding HCC of non-viral etiology, conditions such as the presence of NAFLD [76], obesity [77] or diabetes [78], among others, have been described as risk factors for HCC development and in which increased hepatic activation of NF-κB has also been reported [78,79,80,81].

### 5.2. NF-κB in Kupffer Cells (KCs): Inflammation

Activation of NF-κB is necessary for the development of the innate immune response, being essential for the activation and function of the different cells of the hepatic immune system that participate in this response, including mast cells, dendritic cells and KCs [82]. When NF-κB is activated in these cells, an increased expression of cytokines and chemokines occurs, with the aim of recruiting other cells of the immune system, such as neutrophils, leading to the development of an inflammatory response [82]. Although, activation of KCs results of vital importance in the liver response to infection or damage, the end of this activated state is of equal importance, since its non-resolution leads to the development of an uncontrolled inflammatory process and, therefore, to the possible generation of an inflammatory liver disease [83].

Thus, various studies confirm that regulation of the functional phenotype of KCs (from a normal “tolerogenic” condition to a pro-inflammatory activated state) is associated with the progression of various liver pathologies, including alcoholic liver disease (ALD) [84], NAFLD [85], NASH [86], the development of fibrosis [87] and HCC [88], among others. A proof of the relevance of KCs and the activation of NF-κB in the generation of liver disease has been reflected in the publication by Son et al. [89]. This work shows in livers of animals that had been treated with the toxic CCl_4_ that selective inactivation of NF-κB, mainly in macrophages with an NF-κB decoy, reduced liver damage and fibrosis. Accordingly, we have been able to corroborate in a mouse model of acute liver inflammation induced by LPS that the increase of certain pro-inflammatory cytokines, such as MCP1 (monocyte chemotactic protein, also known as CCL2) and IL6, was markedly reduced by liposomal chlodronate administration, capable of killing KCs [8]. 

KCs can be activated by various types of stimuli, such as pathogen-associated molecular patterns (PAMPs) and damage-associated molecular patterns (DAMPs), from microbial antigens or damaged hepatocytes [82,88]. Thus, due to the wide repertoire of signals that KCs are able to identify and integrate, they will respond differently depending on the tissue microenvironment. Among these multiple responses, it is worth highlighting the activation of the TLR4 by LPS. LPS or endotoxin, a typical PAMP, triggers an NF-κB-dependent response that is the leading cause of inflammation in various types of liver injury, including endotoxemia, alcoholic liver injury, I/R injury, and systemic viral infection [87]. The KCs have a key role in the elimination of endotoxins in the liver and also in the inflammatory response to LPS through the secretion of cytokines such us TNF-α, IL1β, IL6, IL18, IL12 or IL10 [90]. Some of these cytokines contribute to hepatocyte injury, such as the pro-inflammatory TNF-α or IL1β. KCs, hepatocyte and HSCs secrete chemokines, such as MCP1, that promote recruitment of other monocytes/macrophages into the liver, which amplify the inflammatory response initiated by KCs, secreting inflammatory cytokines and ROS and promoting the progression of liver injury [88]. In addition, macrophages (including KCs) not only stimulate the creation of an inflammatory response, but also lead to a fibrogenic response. KCs release of pro-fibrotic cytokines, such as TGF-β, platelet-derived growth factor (PDGF), connective tissue growth factor (CTGF) or tissue inhibitor of matrix metalloproteinase (TIMPs) and trigger HSCs activation [88]. However, KCs also produce anti-inflammatory and regenerative cytokines to reestablish homeostasis. Among these cytokines IL6, a hepatocyte mitogen that is crucial for liver regeneration [91] and IL10, with potent anti-inflammatory and immunoregulatory effects, are key in restoring hepatic homeostasis [92]. 

Ultimately, the phenotype and functionality of KCs and other liver macrophages will depend on the tissue microenvironment, which will determine the balance between the mechanisms of progression and resolution of tissue injury.

### 5.3. NF-κB in Hepatic Stellate Cells (HSCs): Activation, Survival and Inflammation

Furthermore, the involvement of NF-κB has also been implicated in the activation of HSCs, their survival, and inflammatory response. Under physiological conditions, HSCs are in a quiescent state, being their main function to accumulate vitamin A in the form of lipid droplets [93]. However, in response to noxious stimuli, these cells are capable of rapid activation and transdifferentiation into fibrogenic, contractile and proliferative myofibroblast-like cells [93]. Certain signals, such as the presence of apoptotic bodies derived from damaged hepatocytes and some mediators (ROS, TNF-α, PDGF, IL1β, TGF-β) secreted by KCs, platelets, neutrophils and other cells of the immune or inflammatory system, such as endothelial cells or cholangiocytes, contribute to induce the activation and proliferation of HSCs. Furthermore, an altered extracellular matrix also represents a powerful stimulus for the migration/proliferation of activated HSCs, through the expression of integrins, among other signaling pathways [93]. Once activated, HSCs increase the amount and composition of extracellular matrix, contributing to its deposition in the liver, and leading to fibrosis. Moreover, activated HSCs secrete numerous inflammatory, proliferative and fibrogenic cytokines, in both autocrine and paracrine mode of action. To name a few, these include: TGF-β, capable of exerting a positive feedback effect on HSCs themselves, stimulating their activation and proliferation; MCP1, that favors the infiltration and accumulation of immune cells; IL6, important in amplifying the acute phase response; IL10, an anti-inflammatory and anti-fibrogenic cytokine; and numerous adhesion molecules such as intercellular adhesion molecule 1 (ICAM1) or vascular cell adhesion molecule 1 (VCAM1) that are involved in the immune system cells adhesion at the site where liver damage has occurred [93]. 

Both TGF-β and PDGF are considered as the main cytokines responsible for the activation and proliferation of HSCs. The most potent stimulus in inducing the production of type I collagen and other constituents of the extracellular matrix by HSCs is TGF-β, which is autocrine secreted by HSCs [87], but also paracrine secreted by KCs or platelets [94]. The participation of NF-κB is very important in the pro-fibrogenic role of HSCs, since NF-κB activation by LPS/TLR4 pathway in these cells triggers downregulation of TGF-β pseudoreceptor BAMBI, in this way enhancing TGF-β signaling and cell activation [95]. For its part, PDGF represents the most powerful factor involved in inducing the proliferation, differentiation and migration of HSCs, in addition to promoting production and deposition of collagen [96]. During liver injury, HSCs increase their PDGF generation, and also, upregulate PDGF receptors [93]. Platelets are as well potent producers of PDGF [97]. Downstream pathways of PDGF signaling include factors as activator protein 1 (AP-1) and NF-κB, which regulate the expression levels of certain genes involved in cell division, proliferation, fibrogenesis and apoptosis, including type I collagen, TIMPs, matrix metalloproteases (MMPs), the apoptosis regulator Bcl-2 and the E3 ubiquitin-protein ligase XIAP, among others [96].

Furthermore, NF-κB is a pivotal mediator of HSCs survival during the fibrogenic response, as several studies show. In a dynamic environment, HSCs attempt to restore their quiescent state through molecular interconnections involving reversal of the activated phenotype to a quiescent state, apoptosis and premature senescence [98]. Nevertheless, regarding apoptosis, a resistance of activated HSCs to suffer this phenomenon has been observed through the activation of signaling pathways that involve NF-κB and that induce expression of anti-apoptotic proteins such as Bcl-2 [99]. In fact, treatment with NF-κB or proteasome inhibitors has demonstrated to be able to reduce liver fibrosis in vivo in a murine model of bile duct ligation [100]. 

Finally, and as might be expected, NF-κB also has a role in the induction and secretion of inflammatory mediators in HSCs. Such inflammatory mediators include chemokines such as MCP1 (CCL2), C-C motif chemokine ligand 3 (CCL3), C-X-C motif chemokine ligand 2 (CXCL2) and C-X-C motif chemokine ligand 5 (CXCL5), with capacity to increase the infiltration of inflammatory cells to the liver, such as macrophages, which can interact with HSCs and establish a positive feedback on HSCs activation [41].

## 6. Upstream Regulators of SIRT1

The results of diverse research groups [101,102,103] emphasize the relevance of acting on the SIRT1-NF-κB axis as a possible therapy in the treatment of inflammatory diseases. Numerous mechanisms capable of regulating the activity of SIRT1 have been described, among which are: (1) metabolic regulation by NAD^+^; (2) protein-protein interactions; (3) transcriptional and post-transcriptional modulation; (4) post-translational modifications [104]. In this review, we will focus on some upstream regulators of SIRT1 as possible therapeutic targets for modulating inflammation through the SIRT1-NF-κB axis.

Among different proteins capable of interacting with SIRT1 are positive and negative regulators. Some of the most cited are endogenous activators, such as active regulator of SIRT1 (AROS) nuclear protein, or inhibitors, such as endogenous deleted in breast cancer 1 (DBC1) protein, or Tat protein of the human immunodeficiency virus 1 (HIV-1) [105]. 

Other studies regarding upstream regulators of SIRT1, such as IRF9/PPAR-α, NAMPT/NMNAT, HuR, AMPK, PARP1, CK2 and cathepsins have pointed to these proteins as possible therapeutic targets for the treatment of various inflammatory pathologies, including liver diseases. 

### 6.1. IRF9/PPAR-α

Interferon regulatory factors (IRFs) are a transcription factors family integrated by nine members that regulate the expression of genes implicated in creation of innate and acquired immune responses [106]. IRF9, capable of inducing liver damage in a model of hepatic I/R injury, stimulates p53-mediated apoptosis and inflammation by suppressing SIRT1 expression via interaction with its promoter [106].

Interestingly, it seems that IRF9 is able to increase the expression of PPAR-α target genes through an interaction with its promoter region. This interplay improves insulin sensitivity and reduces inflammation and steatosis in obese mice [107]. At the same time, SIRT1 is a target gene of PPAR-α. In heart, the suppression of SIRT1 expression by PPAR-α has been shown to cause cardiac dysfunction [108] by compromising mitochondrial biology and energy homeostasis, which could be palliated with the use of the activator of SIRT1, resveratrol. For this reason, the IRF9-SIRT1 axis could be a promising therapy in treatment of liver inflammation and injury in some hepatic pathologies. However, further investigation is necessary in order to determine the specific participation of the IRF9-SIRT1 axis in liver, and also in order to avoid undesired effects in other organs. 

### 6.2. NAMPT/NMNAT

The NAD^+^ synthesis is mainly carried out from five precursor molecules and intermediate compounds: tryptophan, nicotinamide, nicotinic acid (NA), nicotinamide riboside (NR), and nicotinamide mononucleotide (NMN) [1]. In mammals, the salvage pathway of NAD^+^ biosynthesis from nicotinamide is performed by two enzymes: nicotinamide phosphoribosyltransferase (NAMPT) and nicotinamide mononucleotide adenyltransferase (NMNAT) [109]. 

The first one, NAMPT, the rate-limiting enzyme in the NAD^+^ biosynthetic pathway, is the catalyst for the synthesis of NMN from nicotinamide and 5-phosphoribosyl pyrophosphate (PRPP). The second one, NMNAT, converts the NMN to NAD^+^ [110]. These enzymes, due to their involvement in NAD^+^ synthesis, are able to directly regulate SIRT1 activity and, therefore, they can affect processes in which SIRT1 has shown participation such as glucose tolerance, lipid metabolism or inflammatory response, among others.

One example of this is represented by Wang et al. [111], in which they demonstrate that inhibition of NAMPT aggravates the high fat diet (HFD)/oleic acid induced hepatic steatosis via suppression of SIRT1-mediated signaling pathway. Moreover, administration of NMN, in a murine model of type 2 diabetes (T2D), restores HFD-induced p65 subunit acetylation of NF-κB and the inflammatory gene response, improving also hepatic insulin sensitivity, via SIRT1 activation [112]. Similarly, the study by Caton et al. [113] has reported that NMN administration has anti-inflammatory effects in pancreas and improves insulin secretion in a mice model of diabetes fed with fructose-rich diet and that these beneficial effects are partially blocked by inhibition of SIRT1.

Furthermore, other NAD^+^ precursors, such as NR, have also been tested for the treatment of metabolic and/or inflammatory diseases. When NR enters the cell, it is metabolized into NMN through a phosphorylation step catalyzed by the nicotinamide riboside kinases (NRKs) [114]. Some of the beneficial effects that NR treatment has shown include to enhance the oxidative metabolism and protect against metabolic abnormalities in mice fed a high-fat diet [115] or to attenuate inflammation markers in hepatocytes treated with palmitic acid [116]. 

Finally, and given the promising beneficial effects obtained in murine models through the application of NAD^+^ precursors, some clinical trials for evaluating safety and efficacy of these compounds have also been conducted with healthy volunteers [117] (the results showed that chronic NR supplementation is well-tolerated and elevates NAD^+^ levels) and patients suffering from different pathologies such as Parkinson’s disease [6], cardiovascular disease [118], type 2 diabetes [119] or NAFLD [120,121].

### 6.3. HuR

RNA-binding protein human antigen R (HuR) is a member of the embryonic lethal abnormal-vision family of mRNA-biding proteins that contains three RNA-recognition motifs with capacity of linking to 3′ UTR regions of the SIRT1 mRNA, providing stability to it [122]. 

It is known that levels of HuR decreases significantly with age and that damage to DNA by oxidative stress is able to trigger phosphorylation of HuR by checkpoint kinase 2 (Chk2), a protein kinase activated in response to oxidative stress, causing the dissociation between HuR and SIRT1 mRNA [115]. In this way, a balance is maintained between DNA damage, apoptosis and senescence, so that when significant DNA damage accumulates, the half-life of the *SIRT1* mRNA is reduced by HuR dissociation, thus favoring apoptosis [123].

HuR has been related not only with processes like oxidative stress or apoptosis but also with promoting inflammation. The pro-inflammatory properties of HuR are linked to its interaction with mRNAs encoding pro-inflammatory proteins, being TNF-α and IL6 the most prominent [124]. HuR stabilizes mRNAs of these pro-inflammatory cytokines and enhances their expression in different cell types, including fibroblasts, T-cells and macrophages [124]. However, it has been established that HuR is able to provide stability to *SIRT1* mRNA, fact that could be translated into an anti-inflammatory action of HuR due to the antagonistic relationship between SIRT1 and NF-κB. The study of Wang et al. [125] shows that the relationship between HuR and SIRT1 around the inflammatory response is more complex than might be expected. This manuscript shows that a knockdown of hepatic Slu7, a splicing regulator, ameliorates inflammation and liver injury in a model of alcoholic steatohepatitis in ethanol-fed mice. Reduced Slu7 expression increases the expression of *SIRT1* full-length transcript instead of the alternative splicing *SIRT1-∆Exon 8* isoform induced by ethanol intake and that likely contributes to hyper-acetylation of NF-κB resulting in transcription of NF-κB-dependent inflammatory genes. In addition, it seems that HuR presents binding affinity to exon 8 region of *SIRT1* to stimulate the alternative splicing of *SIRT1* pre-mRNA, and ethanol exposure probably favors the up-regulation of hepatic HuR, thus reinforcing the pro-inflammatory properties of HuR [125].

### 6.4. AMPK

AMP-activated protein kinase (AMPK) is a serine/threonine kinase activated in response to different types of cellular stresses [126]. This protein kinase is activated in response to low energy levels conditions, in front of which it launches the catabolic pathways that lead to generation of ATP, while suppressing anabolic pathways, in which ATP is consumed [126].

Most studies have established a negative association between AMPK and inflammation [127,128,129]. Moreover, various studies [130,131] describe how SIRT1 is a target protein of AMPK, being activated by it. Thus, it is easy to find scientific publications in which researchers have tested different compounds capable of activating the metabolic pathway of AMPK-SIRT1 as a therapeutic target for the treatment of inflammatory liver diseases. The studies conducted by Chyau et al. [132], for example, used antrodan, a purified β-glucan from the fungus species *Antrodia cinnamomea*, with potent hepatoprotective, anti-inflammatory, hypolipidemic and anti-metastatic effects, among others, to treat fatty liver disease in a murine model fed with a high-fat and high-fructose diet. The results showed antrodan alleviated the HFD-induced NAFLD via the AMPK/SIRT1/PPAR-γ pathway. Antrodan, together with adiponectin, induced conversion of AMPK into pAMPK, which, in turn, increased the ratio of NAD^+^/NADH and upregulated SIRT1. This suppressed the hepatic insulin resistance, the level of triglycerides and de novo lipogenesis, while enhanced β-oxidation, in addition to palliate, partially, the inflammatory cell infiltration and the spotty focal necrosis at histological level. 

Similarly, Nagappan et al. [133] administered cryptotanshinone, a diterpene originating from the plant *Salvia miltiorrhiza*, to mice exposed to chronic alcohol feeding in order to evaluate anti-oxidative, anti-fibrotic and anti-inflammatory properties of the compound in ethanol-induced liver injury. The treatment with cryptotanshinone ameliorated ethanol-promoted hepatic steatosis and inflammation, in addition to enhancing the expression of anti-oxidant genes. These effects are attributable to the increase AMPK phosphorylation, triggered by the cryptotanshinone, that seems to be responsible for the rise of phosphorylation and activation of SIRT1. 

### 6.5. PARP1

The poly[ADP-ribose] polymerase (PARP) family is composed by proteins implicated in processes such as DNA damage response, cell death, cell cycle regulation and telomerase regulation [134]. Inside this family, PARP1, a NAD^+^-dependent nuclear ADP-ribosyltransferase, is rapidly activated under pathophysiological conditions in front of which it regulates the expression of disease-related genes (as chemokines or pro-inflammatory mediators) via three mechanisms: (1) chromatin modulation; (2) transcriptional regulation and (3) RNA regulation [135]. 

Both enzymes, PARP1 and SIRT1, use NAD^+^ as a cofactor in their catalytic activity. PARP1, once activated, tends to maintain its activated status for a long time during which it can reduce NAD^+^ intracellular levels significantly. In fact, a correlation has been established between downregulation of NAD^+^ by PARP1 and a decrease in SIRT1 activity level [128]. Conversely, activation of SIRT1 reduces PARP1 activation [136]. But this antagonism between both enzymes is more complex than the simple competition for the same molecule needed as a cofactor for their enzymatic activity. PARP1 and SIRT1 exert completely opposite regulatory effects over the same target proteins: while SIRT1 can activate PGC-1α and FOXO [137], involved in regulation of mitochondrial biogenesis and oxidative metabolism, PARP1 acts suppressing the activities of both transcription factors [137,138].

There are multiple examples of pharmacological modulation of PARP-SIRT1 axis, in any of the two directions of the axis [139,140,141]. Focusing on the regulation of PARP1, numerous inhibitors have been developed to treat cancer [142], cardiovascular disorders [143], metabolic disorders [144] and autoimmune diseases [145], among other pathologies. Regarding the treatment of chronic inflammation and liver diseases, pharmacological inhibition of PARP or genetic deletion of PARP1 was able to reduce chronic inflammation and fibrosis induced by the treatment with CCl_4_ in mice [146], and also attenuated the development of hepatic fibrosis, bile duct ligation-induced, in mice [146].

Moreover, treatment with an inhibitor of PARP1 in mice fed with a high-fat high-sucrose (HFHS) diet reversed NAFLD through repletion of NAD^+^ [147]. The incorporation of PARP1 inhibitor to the HFHS diet increased mitochondrial biogenesis and β-oxidation in the liver, in addition to reduce ROS, endoplasmic reticulum stress, inflammation and fibrosis. Furthermore, using a hepatocyte specific SIRT1 knockout mouse, it was shown that previous benefits found in mice fed with HFHS diet supplemented with a PARP inhibitor were SIRT1-dependent [147].

### 6.6. CK2

Protein kinase CK2 is a constitutively expressed serine/threonine kinase which regulates many cellular functions, including gene expression, translation, cell cycle progression and survival [148]. Also, the role of CK2 has been well characterized in signaling pathways involved in regulating inflammatory responses [149]. In fact, CK2 activity promotes NF-κB activation acting at multiple levels of its activation cascade, targeting not only the subunit p65 of NF-κB itself, but also the upstream regulators of NF-κB: inhibitor of κB (IκB) and IKK complex [150]. For example, the treatment of fibroblasts and hepatoma cells with IL1β leads to activation of NF-κB via phosphorylation of p65 subunit by CK2.

Anomalous CK2 signaling has been associated with development of numerous diseases with inflammatory course, such as glomerulonephritis, atherosclerosis or cancer [151]. A good example of this is represented by the study of Roelants et al. [152]. They observed an upregulation of the CK2 catalytic subunits in samples from renal cell carcinoma (RCC) tumors that did not correlate with the amount of mRNA of these subunits present in most of the samples. Additionally, the positive results that they obtained from the use of a CK2 inhibitor, in vitro, on a hypertriploid RCC cell line revealed this strategy as promising for the treatment of this pathology. Another interesting example is the one offered by the study of Choi et al. [153] in which they not only show the participation of CK2 in inflammatory pathologies, such as obesity and NAFLD, but also identify the obesity-linked Ser-164 phosphorylation of SIRT1 by CK2 as the main responsible mechanism for inhibiting the nuclear localization of SIRT1 and for affecting, to some extent, its enzymatic activity. On the other hand, the report by Shu et al. [154] proves that in vitro stimulation with TNF-α on vascular smooth muscle cells (VSMC) acts enhancing the axis CK2-SIRT1-smooth muscle 22α (SM22α), an actin-associated protein suppressor of NF-κB signaling cascades in VSMCs, which limits the inflammatory response in these cells and supposes a promising therapeutic pathway in preventing cardiovascular diseases. Thus, these studies reveal the complex roles of CK2 in the pathogenesis of inflammatory diseases and evidence the need to further research in order to evaluate the possibility of using CK2-SIRT1 axis as therapy for a future possible treatment of inflammatory liver diseases. 

### 6.7. Cathepsins

Cathepsins are a large and varied group of lysosomal proteases involved in the development of numerous physiological functions such as bone resorption, innate immunity, apoptosis, angiogenesis, and aging, among others [155,156]. Due to this, any deregulation in cathepsins entails the development of numerous pathologies such as arthritis, periodontitis, pancreatitis, obesity, metastasis, stroke or Alzheimer’s disease [155,156]. 

Although one of the mechanisms for the regulation of these enzymes is based on the compartmentalization of them within the lysosomes, several authors have described the existence of mechanisms involved in lysosomal permeabilization as a normal process mediating apoptosis induced by diverse stimuli (oxidative stress, TNF-α or TRAIL cytokines, sphingosines, etc.) [157,158].

Moreover, various research groups, including ours [8], have demonstrated the proteolytic rupture of SIRT1 by different cathepsins. Chen et al. [159] show in their study that SIRT1 is degraded in embryonic progenitor endothelial cells subjected to different types of stress and in which lysosomal permeabilization seems to occur. In addition, they also observe an in vitro proteolytic processing of SIRT1 by cysteine cathepsins B, S and L. Oppenheimer et al. [160] have also described a proteolytic processing of SIRT1 by cathepsin B, to generate a 75 kDa fragment in human osteoarthritic chondrocytes exposed to TNF-α, with a relevant role in the survival of these cells after inflammatory/apoptotic stimulation. 

**Figure 1:**
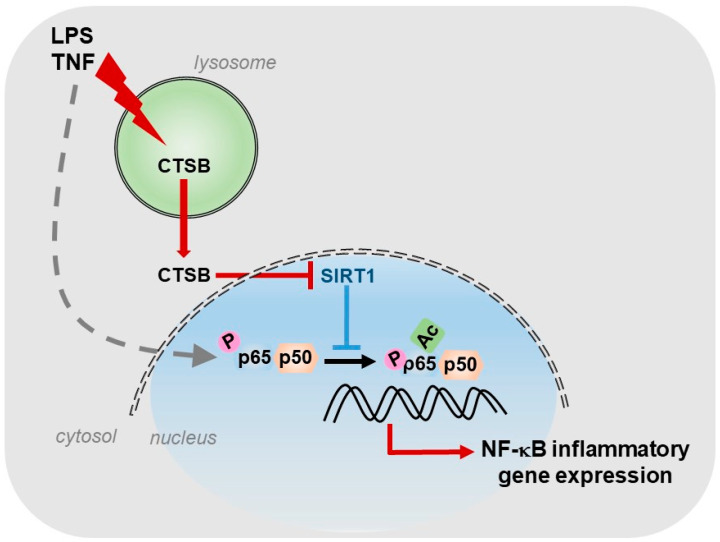
Role of CTSB, in response to inflammatory stimuli, in promoting NF-κB-dependent inflammatory gene expression by inhibiting SIRT1 activity.

For our part, we described the existence of a cathepsin B/S-SIRT1 axis able to control inflammation (Figure 1), in murine models of acute and chronic hepatic diseases, through its actuation over the key transcription factor in regulating inflammation: NF-κB [8]. In our study, we observed, in vitro, a progressive increase in the expression of cathepsins B and S during the activation of HSCs, the main cells responsible for the generation of fibrosis in the liver. Furthermore, towards the end of the activation process of HSCs, we could observe that SIRT1 expression levels decreased considerably with respect to the initial stages of HSCs activation, coinciding with the maximum peak of cathepsins expression and suggesting a possible proteolytic processing of SIRT1 by cathepsins. This was further corroborated by using a cathepsin B inhibitor, Ca074-Me, and a cathepsin S inhibitor, Z-FL-COCHO, and whose presence was sufficient to upregulate the protein level of SIRT1. Furthermore, the inhibition of cathepsins B and S decreased the induction of inflammatory genes, induced by LPS/TNF treatment and dependent on NF-κB, in HSCs [8].

Since different cell types participate in the inflammatory process, we decided to investigate the expression and activity levels of cathepsins B and S, and of SIRT1 not only in HSCs but also in hepatocytes and macrophages, in vitro, using primary cells and cell lines. We observed that macrophages, in general, had the highest level of cathepsin B enzymatic activity. Of interest, this high level cathepsin B activity coincided with the presence of processed isoforms of SIRT1 protein, corresponding to two peptides of 75 and 35 kDa, instead of the full and active 110 kDa isoform, which predominated in the other cell types. Therefore, our results suggested that, in macrophages, SIRT1 processing correlated with the highest degree of cathepsin B enzymatic activity present in these cells and thus, with its potential inflammatory phenotype. Additionally, and as we expected, the use of cathepsins B and S inhibitors was able to reduce in vitro the induction of certain inflammatory genes, NF-κB- dependent, induced by LPS treatment in macrophages [8].

Furthermore, we obtained similar results in vivo. The models of acute inflammation, induced by LPS and of inflammation with presence of liver fibrosis, induced by LPS and CCl_4_, allowed us to corroborate the results obtained with the use of cathepsins B and S inhibitors in a more complex system than the one developed in vitro. In both models, LPS treatment induced the expression of inflammatory genes encoding MCP1 and IL6, NF-κB dependent, which was significantly reduced with the use of cathepsins B and S inhibitors. Treatment with cathepsins inhibitors also potentiated SIRT1 activity, independently of LPS treatment, in both models, correlating increased SIRT1 activity with decreased expression of NF-κB-dependent inflammatory genes. Furthermore, administration of the cathepsin B inhibitor, Ca074-Me, in the acute inflammation model LPS-induced, decreased liver damage generated by LPS treatment [8], underscoring the lysosomal permeabilization induced by LPS/TNF [161] and the role of cathepsin B released to the cytosol, not only at the inflammatory level, but also in hepatocyte apoptosis [162]. In addition, cathepsin B plays a relevant role in HSCs activation and fibrogenesis. For instance, after chronic CCl_4_ administration, cathepsin B expression increased in HSCs but not in hepatocytes, while its inactivation reduced inflammation and collagen deposition [163] via an acid sphingomyelinase-dependent mechanism [164].

In summary, our results reveal the regulation that cathepsins B and S exert on the hepatic inflammatory gene expression, NF-κB-dependent, through its inhibitory action on SIRT1. This opens the door to the use of cathepsins inhibitors as one more possible therapeutic strategy for the treatment of inflammatory liver diseases.

## 7. Other Modulators of SIRT1: From Resveratrol to STACs (Sirtuin Activating Compounds)

**Figure 2:**
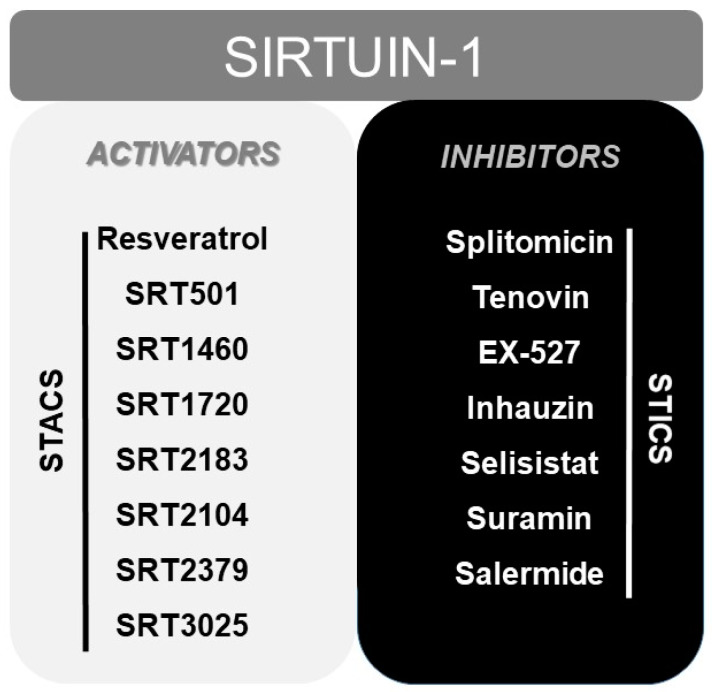
Scheme displaying the most typical activators (STACS), and inhibitors (STICS) of SIRT1.

Finally, numerous pharmacological or natural (like resveratrol [165]) modulators of SIRT1 have been reported to date, including sirtuin activating compounds (STACs, such as SRT1460, SRT1720, SRT2183 or SRT2104) and sirtuin inhibiting compounds (STICs, such as splitomicin, tenovin or EX-527) [166] (Figure 2).

These STAC were investigated and designed as a result of the beneficial outcomes obtained, at health level, in numerous studies in which overexpression of sirtuin was used in murine models [167,168]. In fact, some of these synthetic STACs above mentioned, with much more potency than resveratrol, have reached phase I and II of clinical trials, including SRT2104, SRT2379 and SRT3025 [169] (www.clinicaltrials.gov). Even resveratrol and SRT501 (a micronized resveratrol formulation) have reached phase IV of clinical trials. However, clinical trials with resveratrol showed inconsistent beneficial effects between different trials [169], poor bioavailability [170] and problems derived from its simultaneous activity on multiple targets [171,172]. In this sense, although SRT2104, SRT2379 and SRT3025 have demonstrated a good tolerability in clinical trials with healthy volunteers, only SRT2104 showed some beneficial effects in both healthy volunteers and in patients. SRT2104 proved to improve the lipid profiles, although not the glucose and insulin control, of patients with diabetes mellitus Type 2 [173]. It also demonstrated to reduce serum cholesterol, LDL levels and triglycerides level in healthy cigarette smokers [174]. More important, SRT2104 was able to reduce LPS-induced IL6 and IL8 cytokines release after single and repeated administration in healthy volunteers [174]. 

Thus, further investigation on finding new SIRT1 activators or on improving current ones is needed. But, it is also important to consider the need to investigate about SIRT1 activation at the multi-organ level. SIRT1 is expressed in a wide variety of tissues, including metabolically active tissues, such as liver, skeletal muscle, pancreas, adipose tissue, or brain, where it develops a wide range of physiological processes [14]. For this reason, application of SIRT1 activators at systemic level should be carefully studied, in order to avoid undesired effects due to activation of SIRT1 in multiple organs. An example of this is that systemic SIRT1 activation promotes lipolysis in adipocytes, with the consequent release of large amount of free fatty acids to circulation, that finally arrive to the liver, inducing or aggravating a possible steatosis [12].

## 8. Other Considerations: Anti-Oxidant Properties of SIRT1

Another fact to keep in mind when talking about modulation of SIRT1 as anti-inflammatory therapy for the treatment of liver diseases, specially metabolic ones, is the anti-oxidant properties attributed to SIRT1 and other sirtuins (through deacetylation of transcription factors, such as FOXO family or PGC-1α, with capacity to induce expression of numerous anti-oxidant enzymes) [175]. These properties could be beneficial because of the often-existing correlation between the presence of metabolic diseases and an impairment of the antioxidant defense system in the liver and other organs. For example, the presence of NAFLD in insulin-resistant subjects has been correlated with existence of severe stress oxidative and endothelial dysfunction, independently from the presence of metabolic syndrome or adiposity [176]. At the same time, oxidative stress is associated with endothelial dysfunction and cardiovascular disease, and in NAFLD patients, it triggers an inflammatory response and deposition of extracellular matrix in the liver, favoring the development of NASH [177]. 

Moreover, there is evidence that oxidative stress and inflammation, processes that are interconnected, are linked to senescence and aging. On the one hand, it seems that cells, during their lifetime, gradually lose their ability to defend themselves from oxidative stress, with the consequent accumulation of this over time [178]. On the other hand, it seems that as humans age, there is a remodeling of the immune system that leads to the establishment of a chronic inflammatory state (inflammaging) due to continuous exposure to different types of endogenous and environmental stress [179]. Because of this, it would also be interesting to explore the benefits of SIRT1 modulators in age-associated diseases, which share an inflammatory base and high levels of oxidative stress, such as COPD (chronic obstructive pulmonary disease) or Alzheimer’s disease [180].

Finally, it would be interesting to note the differential effects of SIRT1 (pro-apoptotic or cytoprotective) at the hepatic level, depending on the liver damage model being studied and the consequences derived from a potentiation of SIRT1 taking into account these differences. For example, publication of Farghali et al. [181] shows that in an in vivo acute model of chemically induced hepatotoxicity by a single-dose treatment with D-galactosamine/lipopolysaccharide (D-GalN/LPS) there is a downregulation of SIRT1 expression. In this model, the compensatory anti-oxidant response generated to alleviate possible damage caused by the release of ROS and other cytotoxic/inflammatory mediators by the immune system proves to be insufficient (at least at the doses used in the study) and significant liver damage occurs. This decompensated ROS production can negatively affect SIRT1 activity and expression level. Treatment of animals, prior to D-GalN/LPS administration, with some STACs (quercetin and SRT1720) is sufficient to upregulate SIRT1 levels and reduces D-GalN-LPS-induced hepatoxicity. However, in an in vivo chronic model of chemically induced hepatotoxicity by repeated doses treatment with carbon tetrachloride (CCl_4_) in which there is also an important liver damage and oxidative stress production by immune system cells and derived from CCl_4_ metabolism, there is an upregulation of SIRT1 expression. In this case, authors hypothesize that persistent oxidative stress induces SIRT1 expression via JNK-dependent FOXO1 activation and that elevated levels of ROS, as reported on CCl_4_-induced model of liver injury, can initiate SIRT1-mediated apoptosis (through p53). This could be a good explanation of why, in this case, when a STAC (quercetin) is administered repeatedly and in parallel with the administration of CCl_4_, the liver damage produced in this model is mitigated even though quercetin reduces the expression of SIRT1. So, it seems that quercetin is able to fine-tune SIRT1 expression to a lower but still effective level to deal with xenobiotic-induced hepatotoxicity.

## 9. Conclusions

Sirtuin 1 (SIRT1) is a class III histone deacetylase whose catalytic activity is dependent on NAD^+^ cellular availability and that, therefore, can act as metabolic sensor. It has been reported high levels of expression of SIRT1 in metabolic tissues such as liver, adipose tissue, brain, muscle or pancreas. In liver, SIRT1 is crucial for glucose, lipids and cholesterol homeostasis. Consequently, it has been described the participation of SIRT1, and other sirtuins, in some metabolic diseases, NAFLD (non-alcoholic fatty liver disease) among them, both in animal models and humans.

The nuclear factor kappa B (NF-κB) is a family of inducible transcription factors present in numerous cell types and whose main heterodimer is composed by p65/p50 subunits. It is considered as a master regulator of the inflammatory response due to its capacity to regulate transcription of genes involved in immune and inflammatory response. 

Both, SIRT1 and NF-κB signaling pathways have evolved jointly for the maintenance of homeostasis, allowing that body energy status to be coupled with inflammatory and immune response in front of a possible noxious stimulus. However, the nature of this relationship is antagonistic and, importantly, a direct association between SIRT1 and NF-κB has been established, in which SIRT1 is able to deacetylate p65 subunit of NF-κB, triggering the transport of NF-κB from nucleus to cytoplasm and, therefore, suppressing its transcriptional activity. Interestingly, some authors have established a possible association between the antagonistic relationship of SIRT1 and NF-κB in the hypothalamus, a central regulator in maintaining the metabolic energy balance in the body, and the generation of metabolic dysregulations in peripheral body tissues as potential sources of metabolic diseases [43]. For future studies, it would be interesting to explore whether NF-κB activation in the hypothalamus, by over-nutrition, is accompanied by a decrease in SIRT1 and whether it may be the cause of metabolic dysregulation in other organs, thus leading to metabolic diseases. 

Diverse research groups have emphasized the relevance of SIRT1-NF-κB axis as a possible therapeutic target for treatment of inflammatory diseases. In relation to this, it is necessary to highlight that several upstream regulators of SIRT1 have been described, including IRF9, PPAR-α, NAMPT, NMNAT, HuR, AMPK, PPAR1, CK2 and cathepsins, which are able to regulate inflammation through SIRT1 modulation and that, as a consequence, have been involved in different inflammatory disorders, including hepatic diseases. Also, some natural (resveratrol) or synthetic compounds (STACs) with capacity to activating SIRT1 have been discovered and used to treat inflammatory diseases, some of them even reaching I and II phase of clinical trials. 

In addition, and due to known association between senescence and aging with oxidative stress and inflammation, age-associated diseases could also benefit from the use of STACs. Of course, and in view of the differential effects that SIRT1 can trigger depending on the nature and type of liver damage present (acute or chronic), further research will be needed on studying the possible benefits/harms that may result from the use of SIRT1 activators/inhibitors in each setting. While NF-kB is considered a master regulator of inflammation in hepatic diseases, at the same time it can induce a pro-survival response in hepatocytes, the main cell type present in the liver [41]. Therefore, it is important to consider learning about the effect that these SIRT modulators have on the different types of cells present in the liver, and not just inflammatory ones, and/or the use of the targeted delivery of these agents to specific cell types, to better address their safe therapeutic use in liver diseases.

Given that the burden of liver pathologies throughout the world supposes numerous deaths every year and that, until now, there is no effective cure for many of these pathologies, whose common feature is usually the presence of inflammation to a greater or lesser extent, approaches attempting to target the mechanisms that modulate inflammation, such as the SIRT1-NF-κB axis, could represent a major breakthrough in the treatment of liver disease.
